# UPLC-MS/MS method for Icariin and metabolites in whole blood of C57 mice: development, validation, and pharmacokinetics study

**DOI:** 10.3389/fphar.2023.1195525

**Published:** 2023-07-20

**Authors:** Wei Liu, Xiuyun Li, Na Li, Ze Mi, Na Li, Jinjing Che

**Affiliations:** ^1^ State Key Laboratory of Separation Membranes and Membrane Processes/National Center for International Joint Research on Separation Membranes, Tiangong University, Tianjin, China; ^2^ Beijing Institute of Pharmacology and Toxicology, State Key Laboratory of Toxicology and Medical Countermeasures Beijing, Beijing, China; ^3^ Center of Drug Evaluation, National Medical Products Administration, Beijing, China

**Keywords:** IcariinIcariside I, Icariside II, serial whole blood sampling, UPLC-MS/MS, pharmacokinetics

## Abstract

Icariin, a Chinese medicinal herb with significant effects on Alzheimer’s disease, lacks pharmacokinetic data in mice. To address this, a UPLC-MS/MS method was developed and validated for quantifying Icariin and its metabolites, Icariside I and Icariside II, in the whole blood of mice. The method processed micro-whole blood from serial collections of the same C57 mouse, with well-fitted linearity (0.25–800 ng mL^−1^) and intra- and inter-day precision and accuracy within 15%. Short-time and autosampler stability were verified, with acceptable extraction recoveries and matrix effects over 74.55%. After intravenous administration (15 mg kg^−1^) of Icariin in C57 mice, Icariside I and Icariside II were detected within 2 min. However, after the intragastric administration (30, 90, and 150 mg kg^−1^) of Icariin in C57 mice, Icariin and Icariside I were not detected, and Icariin was rapidly converted into Icariside II. Furthermore, the C_max_ and AUC_0-t_ of three doses (30, 90, and 150 mg kg^-1^) of Icariside II increased as the dose increased. In conclusion, this method improves the traditional method of collecting only one blood sample from each mouse, detecting Icariin and its metabolites in the whole blood of mice, especially for serial collection of micro-whole blood.

## Graphical Abstract

**Figure:**
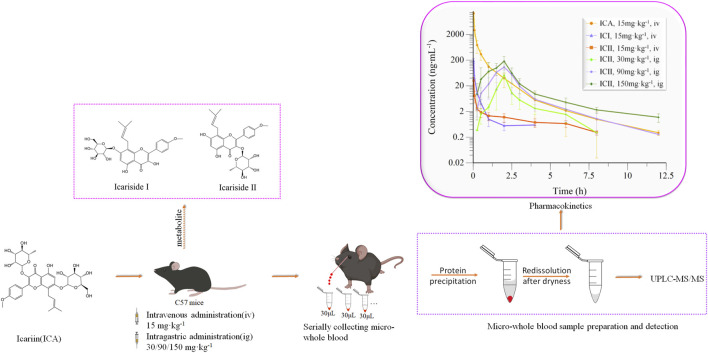
Pharmacokinetics study of Icariin and Metabolites in Whole Blood of C57 Mice.

## 1 Introduction

Icariin (ICA) is a major constituent of flavonoids that are isolated from Epimedium brevicornum Maxim (Jin et al., 2019). ICA has been reported to exhibit numerous pharmacological activities, including anti-oxidant, anti-inflammatory, and anti-apoptotic effects, which may contribute to the preventive and/or therapeutic benefits of Icariin in various disorders in the nervous system, such as cerebral ischemia, Alzheimer’s disease (AD), Parkinson’s disease, multiple sclerosis, and depression (Wang et al., 2017; Cao et al., 2019; Khezri and Ghasemnejad-Berenji, 2022). ICA has been shown to protect neurons in the central nervous system from degeneration by inhibiting neuronal apoptosis and tau protein hyperphosphorylation, and Icariin is a potential drug for treating Alzheimer’s disease (Angeloni et al., 2019). Previous studies have reported that ICA improves spatial learning and memory in different mouse models, including Aβ (25-35)-treated rats, 10-month-old APP/PS1 transgenic mice, APP transgenic (Tg) mice, Tg2576 mice, and triple-transgenic (3×Tg) mice (Jin et al., 2019; Wang et al., 2021; Yan et al., 2023) C57 mice participated in this experiment as background mice of APP/PS1 transgenic mice.

**FIGURE 1:**
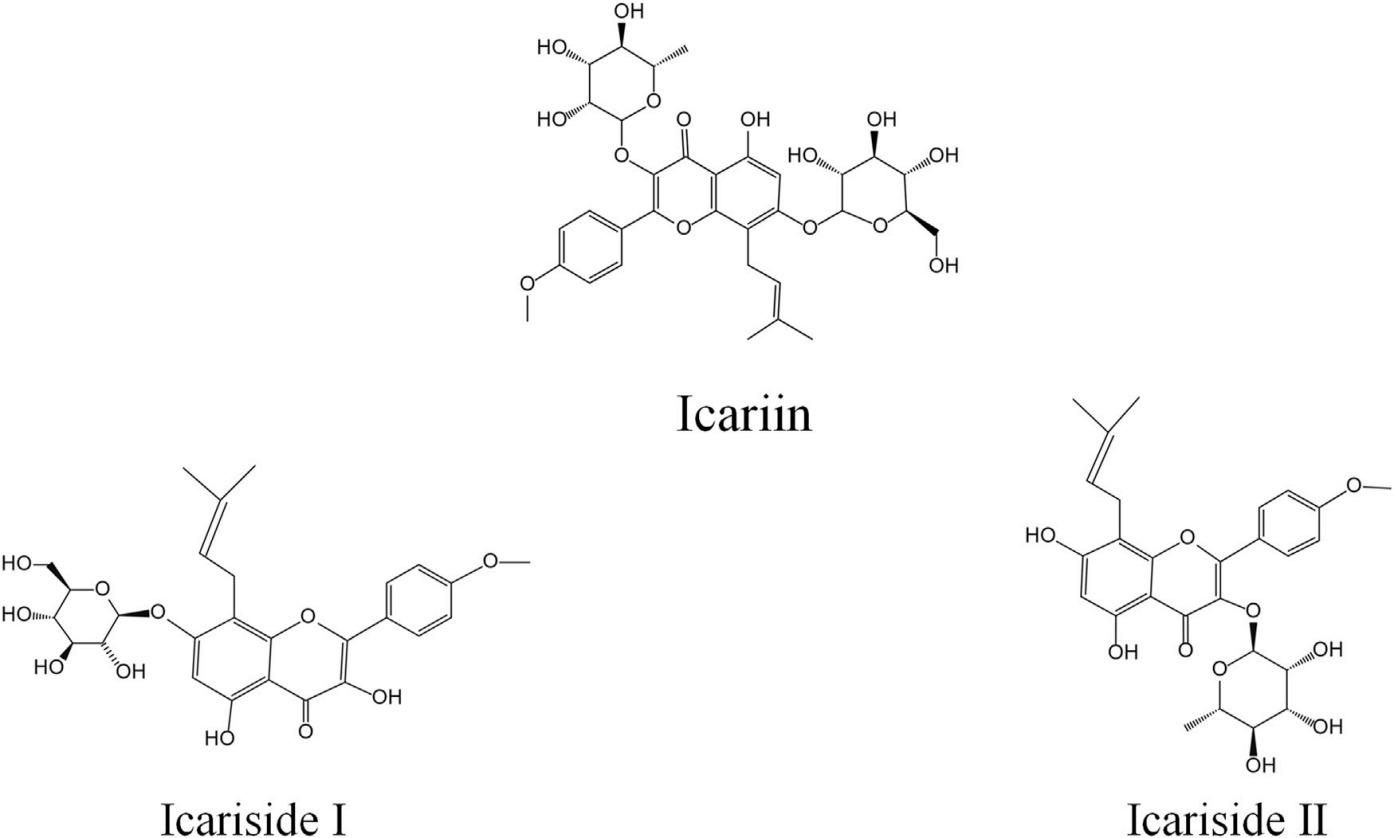
Structural formula of Icariin, Icariside I and Icariside II.

Metabolic and pharmacokinetic studies have shown that derivatives in rats can be obtained through metabolism, by converting Icariin to Icariside I (ICI) and Icariside II (ICII), whose structural formulas are shown in Figure 1 (Wang et al., 2020). ICI is produced by the loss of rhamnose at ICA B-ring position 3, and ICII is produced by the loss of glucose at the position 7 of the A-ring of ICA (Xu et al., 2017). However, our previous study showed that ICA may be metabolized primarily to ICII by bacteria in the rat intestine (Xu et al., 2017). As the predominant bioactive form of ICA *in vivo*, ICII’s pharmacological properties are similar to ICA, including a strong biological resistance to inflammation, osteoporosis, hypoxia, cancer, and erectile dysfunction (Wang et al., 2018; Jin et al., 2019; Zeng et al., 2022; Zhang et al., 2022), and ICII effectively ameliorates cognitive function deficits, inhibits neuronal degeneration, and reduces platelet burden formation, which may contribute to AD therapy (Yan et al., 2017).

Appreciation of the pharmacokinetic properties of ICA is critical to understanding its pharmacological effects and potential for pharmacological development. Methods developed using capillary zone electrophoresis and high-performance liquid chromatography (HPLC) can only be applied to the quantitative determination of Icariin and its metabolites in biological samples. These methods are time-consuming and not sensitive enough for quantitative determination (Chen et al., 2007; Chen et al., 2009; Wang et al., 2015). Several high-performance liquid chromatography-tandem mass spectrometry (LC-MS/MS) methods have been developed for the detection and quantification of ICA and its metabolites (Cheng et al., 2015). To study the pharmacokinetics of Icariin in rats, Xu used LC-MS/MS to detect ICA, ICI, and ICII in rat plasma with the limit of detection of 1, 0.5, and 0.5 ng·mL^−1^, respectively, and linearity of 1–1,000, 0.5-1,000, and 0.5–1,000 ng·mL^−1^ respectively (Xu et al., 2007), and Liu used capillary zone electrophoresis to detect ICA in rat serum with the sensitivity of 2.5 mg·L^−1^ and linearity of 2.5–150 mg·L^−1^ (Liu et al., 2004).

As far as we know, the pharmacokinetics of ICA were mainly based on rat plasma or rat serum, while pharmacodynamics was mainly studied in mice, and no pharmacokinetic characteristics experiments were conducted in mice. Because the blood volume of the mice is less, about 1.5 mL (Diehl et al., 2001), data on a drug concentration-time profile are required to collect from multiple mice. But the results of this method have great individual differences and cannot accurately reflect the pharmacokinetic characteristics. Alison serially collected blood samples through the tail vein, and the results showed that inter-subject variability across PK parameters was less than 30%, and by serial sampling, there were savings of 40%–80% in study costs, animal use, study length, and drug conservation (Joyce et al., 2014). To overcome this problem, we used protein precipitation that contained redissolution after evaporating to dryness to process micro-whole blood, and this method could use the same mouse to obtain a concentration-time profile.

To study the pharmacokinetics of Icariin, we developed and validated a UPLC-MS/MS method for simultaneous determination of ICA and its two metabolites in the whole blood of mice. This study was the first report on the method of detecting the concentration of Icariin, Icariside I, and Icariside II in the whole blood of C57 mice and the pharmacokinetics of Icariin in C57 mice, especially serial micro-whole blood sampling techniques were used. The analytical method and pharmacokinetic results provided a basis for the study of ICA and its metabolism in APP/PS1 transgenic mice.

## 2 Materials and methods

### 2.1 Materials and animals

Icariin (purity >98%, ICA) was purchased from National Institute for the Control of Pharmaceuticals and Biological Products (Beijing, China). Icariside I (purity >98%, ICI) and Icariside II (purity >98%, ICII) were extracted from Wokai Biotechnology Co., Ltd. (Beijing, China). Daidzein (purity >98%, Dai, IS) was purchased from Yuanye Bio-Technology Co., Ltd. (Shanghai, China). Dimethyl sulfoxide (purity >99.7%, DMSO) was purchased from InnoChem Science and Technology Co., Ltd. (Beijing, China). HPLC-formic acid, HPLC-grade acetonitrile, and HPLC-grade methanol were purchased from Thermo Fisher Inc. (United States). All other reagents and chemicals were of analytical grade.

**FIGURE 2:**
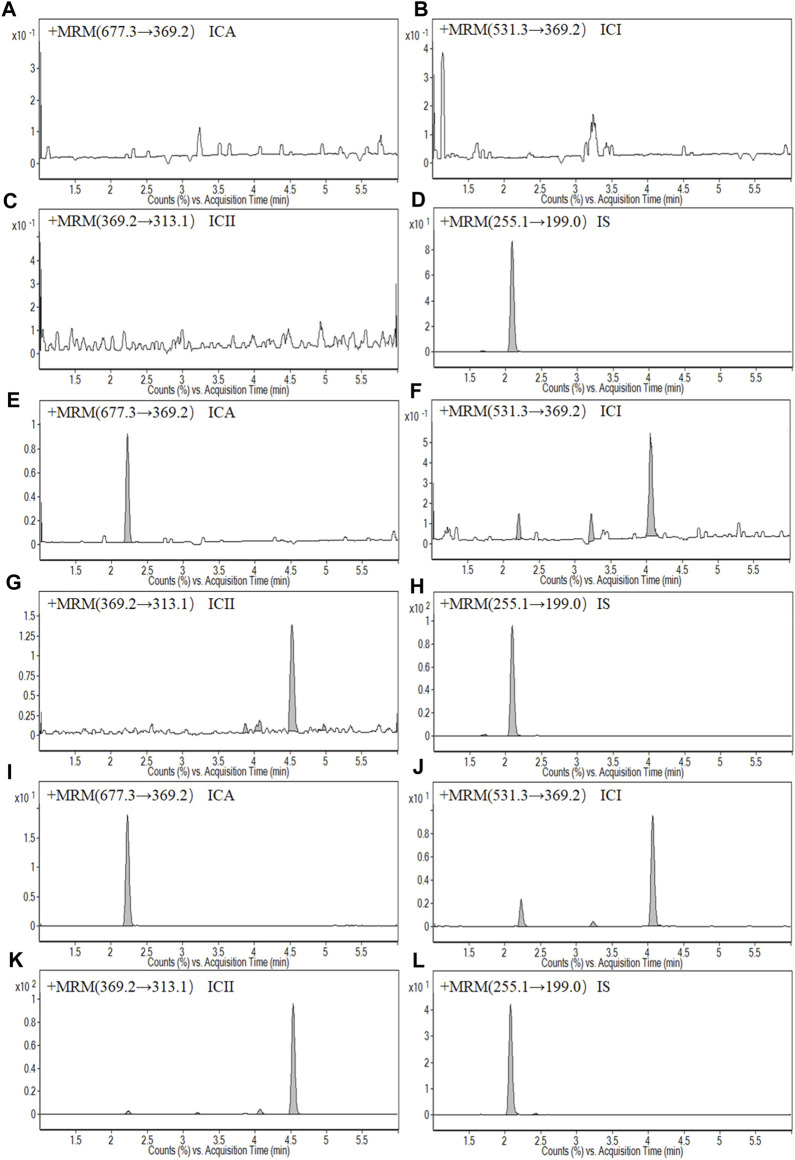
Representative MRM chromatograms: **(A)** A blank whole blood sample of Icariin (ICA); **(B)** A blank whole blood sample of Icariside I (ICI); **(C)** A blank whole blood sample of Icariside II (ICII); **(D)** A blank whole blood sample of Daidzein (IS); **(E)** A LLOQ sample of ICA; **(F)** A LLOQ sample of ICI; **(G)** A LLOQ sample of ICII; **(H)** A LLOQ sample of IS; **(I)** A whole blood sample of ICA at 1.5 h after intragastric administration at a dose of 150 mg kg^−1^ ICA; **(G)** A whole blood sample of ICI at 1.5 h after intragastric administration at a dose of 150 mg kg^−1^ ICA; **(K)** A whole blood sample of ICII at 1.5 h after intragastric administration at a dose of 150 mg kg^−1^ ICA; **(L)** A whole blood sample of IS at 1.5 h after intragastric administration at a dose of 150 mg kg^−1^ ICA.

Male C57 mice weighing 20–22 g each were supplied by Beijing Charles River Laboratory Animal Technology Co., Ltd. (Beijing, China) (Permit number: SCXK (Jing) 2021-0006). All animal procedures were approved by the Ethics Committee and Institutional Animal Care and Use Committee of the Beijing Institute of Pharmacology and Toxicology (Animal Ethics Number: IACUC-DWZX-2021-763). Animal studies were reported in compliance with the ARRIVE guidelines (Percie du Sert et al., 2020). The mice were kept in plastic cages for 24 h, given free pellet food and water, and put on a 12 h light/dark cycle Figure 2.

### 2.2 UPLC–MS/MS instrumentation and analytical conditions

Biological samples were detected using UPLC-MS/MS (AGILENT 1290–6410B, United States), and CAPCELL PAK MG Ⅱ C18 column (2.0 mm · 100 mm, 3.0 μm) for separation. The mobile phase(A) was water containing 0.1% formic acid, and the mobile phase(B) was acetonitrile containing 0.1% formic acid. The linear gradient elution program was as follows: 0.00–5.00 min 25%–60% B; 5.00–5.50 min 60%–85% B; 5.50–5.80 min 85%–25% B. The flow rate was set at 0.4 mL min^−1^ and the injection volume was 5 μL.

The mass spectrometry system equipped with an ESI source. Multiple reaction monitoring (MRM) was selected to quantify in positive ion mode. The optimized parameters of the instrument were obtained as follows: capillary voltage at 4000 V, nitrogen nebulizer gas pressure at 30 psi, gas temperature at 350°C with a flow rate of 10 L·min^−1^. MRM was used to monitor the precursor-to-product ion transitions of m/z 677.3→369.2 for ICA, 531.3→369.2 for ICI, 369.2→313.1 for ICII, and 255.1→199 for Daidzein. The fragmentor energies for ICA, ICI, ICII, and Daidzein were set at 142, 137, 191, and 125 V, respectively. The optimized collision energies of 28, 16, 24, and 25 eV were used for ICA, ICI, ICII, and Daidzein, respectively.

### 2.3 Preparation of standard and quality control (QC) samples

By dissolving the required amount of ICA, ICI and ICII in each DMSO, the standard stock solutions of ICA, ICI and ICII of 2 mg·mL^−1^ were prepared. Daidzein at a concentration of 100 μg·mL^−1^ was used as the internal standard (IS). Calibration curves were prepared for ICA, ICI, and ICII at concentrations of 0.25, 1, 2, 5, 50, 100, 200, 400, and 800 ng·mL^−1^. Additionally, QC samples were prepared at concentrations of 0.5, 20, and 600 ng·mL^−1^ for ICA, ICI, and ICII. All whole blood samples spiked at all levels were stored at 4°C for validation and subject sample analysis.

### 2.4 Sample preparation

**FIGURE 3:**
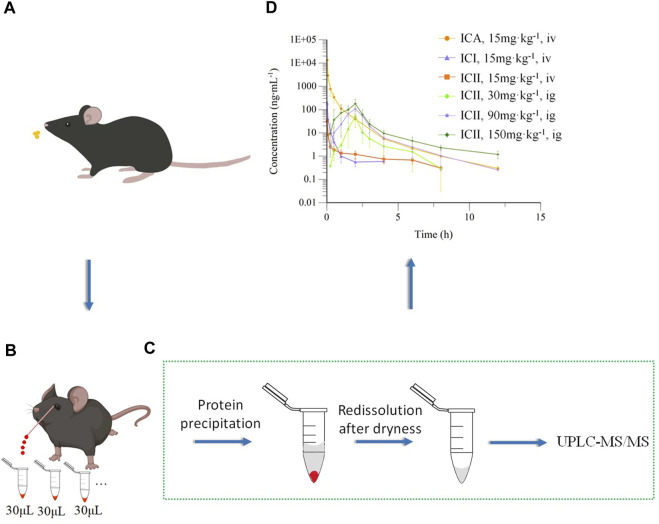
Pharmacokinetics of Icariin in C57 mice. **(A)** Intravenous (15 mg kg^−1^) or intragastric (30, 90 or 150 mg kg^−1^) administration of Icariin in C57 mice; **(B)** Collecting micro-whole blood through capillary from the orbital venous plexus of the same mouse; **(C)** Preparation and detection of whole blood sample; **(D)** Mean drug concentrations of Icariin (ICA), Icariside I (ICI) and Icariside II (ICII) over time in mice after intravenous (iv) or intragastric (ig) administration of ICA (n = 5).

In this study, a protein precipitation method was utilized to extract compounds from whole blood. Specifically, 90 μL of acetonitrile precipitant containing IS at a concentration of 100 ng·mL^−1^ was added to a 30 μL whole blood sample. The samples were then vortex-mixed at 1,500 rpm for 1 min and centrifuged at 16,000×g for 10 min at 4°C. The resulting supernatant was transferred to another tube and evaporated to dryness at 45°C. The samples were then redissolved with 45 μL of 60%:40% methanol:water (v:v), vortexed for 1 min, and centrifuged at 16,000×g for 10 min. The resulting supernatant was injected into the UPLC-MS/MS system. The preparation and detection of the whole blood sample are shown in Figure 3C.

### 2.5 Method validation

#### 2.5.1 Selectivity

The selectivity of the method was investigated by comparing chromatograms of six blank whole blood samples, whole blood samples spiked with ICA, ICI, ICII and IS, and a whole blood sample obtained after intragastric administration of ICA to mice. The chromatograms were examined to determine the presence of any endogenous constituents that could potentially interfere with the analysis of ICA, ICI, ICII and IS.

#### 2.5.2 Linearity and lower limit of quantification (LLOQ)

A linear calibration curve was established by plotting the peak area ratio of ICA, ICI and ICII to IS against the standard concentration (0.25, 1, 2, 5, 50, 100, 200, 400, and 800 ng·mL^−1^) of ICA, ICI and ICII. Least square regression was performed using a 1/(concentration)^2^ weighted linear least square regression model. The criterion for acceptability was a correlation coefficient of the calibration curve that is equal to or greater than 0.99 (R^2^ ≥ 0.99). The LLOQ calibration curve was defined using five lots of blank whole blood samples. The calibration curve must satisfy the following criteria: deviations of no more than 20% around LLOQ (0.25 ng·mL^−1^), and LLOQ is defined as the concentration that produces a signal-to-noise ratio of at least 5.

#### 2.5.3 Precision and accuracy

The intra- and inter-day precision and accuracy of the method were investigated by determining LLOQ (0.25 ng·mL^−1^), LQC (0.5 ng·mL^−1^), MQC (20 ng·mL^−1^) and HQC (600 ng·mL^−1^) samples (five replicates for each concentration level) over three consecutive days. Daily calibration curves were used to calculate the concentrations. The precision of the method at each QC concentration was expressed as the relative standard deviation (RSD), and the accuracy was described as the relative error (RE). The suitability of precision and accuracy was assessed using the following criteria: RSD≤±15% and RE≤±15%.

#### 2.5.4 Extraction recoveries and matrix effects

The extraction recoveries of LQC (0.5 ng·mL^−1^), MQC (20 ng·mL^−1^) and HQC (600 ng·mL^−1^) samples (n = 5) were determined by comparing the peak areas of protein precipitation-extracted samples with the same concentration level dissolved in the processed blank sample [the final solution of blank whole blood after extraction and dissolution with 60%:40% methanol:water(v:v)]. To evaluate the absolute matrix effect of ICA, ICI and ICII, the peak areas of blank whole blood samples after protein precipitation and addition of a compound solution [60%:40% methanol:water (v:v)] containing three QC samples were compared with those of the corresponding QC sample prepared with pure water. Five replicates were evaluated for each of the three different QC concentrations of ICA, ICI and ICII with the same concentration of IS.

#### 2.5.5 Stability

LQC (0.5 ng·mL^−1^), MQC (20 ng·mL^−1^) and HQC (600 ng·mL^−1^) samples (n = 5) of whole blood analytes were prepared and analyzed to evaluate the stability of sample processing. The short-term stability was evaluated by maintaining the QC samples at room temperature (25°C) for 6 h, and autosampler stability was evaluated by placing the extracted QC samples in the autosampler at 4°C for 24 h. The stability of the samples was evaluated by calculating the relative error (RE) between the measured and expected values. As whole blood cannot be stored for a long time, the long-term stability of whole blood cannot be evaluated.

#### 2.5.6 Dilution reliability

Stock solutions of ICA (30 μg·mL^−1^) were prepared. Then, ICA stock solution was further diluted with blank whole blood for 50-fold (v/v). ICA was then extracted as protocol in Section 2.4.

### 2.6 Animal protocol and pharmacokinetic study

Twenty mice were randomly divided into four groups and fasted for 12 h prior to drug administration. The low dose used in this study was based on Jin’s literature (Jin et al., 2014) and the dose is scaled up. These doses provided a basis for subsequent pharmacokinetic experiments in APP/PS1 model mice. One group received a single intravenous dose of ICA solution at 15 mg·kg^−1^, while the other three groups received a single intragastric dose of ICA solution at 30, 90, and 150 mg·kg^−1^. The intravenous ICA solution was prepared by dissolving 1.5 mg of ICA in 6 μL of DMSO, diluting it with 294 μL of 1,2-propanediol, and finally adding 300 μL of water to obtain a 2.5 mg·mL^−1^ solution. The intragastric ICA solution was prepared using 0.05% CMC-Na. Whole blood samples were collected from the orbital venous plexus at 0, 0.03, 0.08, 0.25, 0.5, 1.0, 2.0, 4.0, 6.0, 8.0, and 12.0 h after intravenous administration, and at 0, 0.25, 0.5, 1.0, 1.5, 2.0, 2.5, 3.0, 4.0, 6.0, 8.0, and 12.0 h (except for the 30 mg·kg^−1^ dose at this time point) after intragastric administration. Micro-whole blood samples were collected from the orbital venous plexus of the same mouse in Figure 3B using a capillary. The collected whole blood samples were transferred into heparinized tubes, and 30 µL of whole blood was treated directly according to Section 2.4.

### 2.7 Data analyses

The data of the experiments were presented as the mean ± standard deviation (SD). The experimental data were analyzed by WinNonlin (version 8.1.0, Pharsight, United States) and Excel (version 2019, Microsoft, United States).

## 3 Results

### 3.1 Method validation

#### 3.1.1 Selectivity

Figure 1 shows typical chromatograms of a blank whole blood sample, an LLOQ sample, and a sample collected at 1.5 h after intragastric administration of 150 mg·kg^−1^ ICA. The chromatographs demonstrate selectivity, with retention times of 2.23, 4.06, 4.53, and 2.08 min observed for ICA, ICI, ICII and IS, respectively. Additionally, there was no significant interference from the blank whole blood at the corresponding retention times.

#### 3.1.2 Linearity and LLOQ

The linearity of spiked mice whole blood containing ICA, ICI and ICII was found to be linear in the range of 0.25–800 ng·mL^−1^, with an R^2^ value of ≥0.99. The standard calibration curves for ICA, ICI and ICII exhibited excellent linearity, as shown in Table 1. The lower limit of quantification for the determination of ICA, ICI and ICII in whole blood was defined as the lowest concentration analyzed with an accuracy within ±20% and a precision of ≤20%, which was found to be 0.25 ng·mL^−1^, as shown in Table 1. These limits were sufficient for pharmacokinetic studies of Icariin and its major metabolites following the administration of ICA.

**TABLE 1:** Linearity for ICA, ICI and ICII in whole blood (n = 5).

Analyte	Batch	ICA		Linear range
		Regression equ.	R^2^	
ICA	1	y = 0.0237x+0.0109	0.9914	0.25–800 ng mL^-1^
	2	y = 0.0105x+0.0002	0.9925	
	3	y = 0.0169x+0.0002	0.9940	
	4	y = 0.0196x+0.0021	0.9927	
	5	y = 0.0127x+0.0013	0.9925	
ICI	1	y = 0.0130x+0.0011	0.9933	
	2	y = 0.0095x-0.0011	0.9923	
	3	y = 0.0096x+0.0004	0.9911	
	4	y = 0.0113x+0.0001	0.9928	
	5	y = 0.0081x-0.0003	0.9934	
ICII	1	y = 0.0442x+0.0067	0.9941	
	2	y = 0.0334x+0.0015	0.9918	
	3	y = 0.0333x+0.0048	0.9918	
	4	y = 0.0384x+0.0066	0.9959	
	5	y = 0.0259x+0.0007	0.9901	

#### 3.1.3 Precision and accuracy

Table 2 summarizes the intra-day and inter-day precision and accuracy data for ICA, ICI and ICII from LLOQ (0.25 ng·mL^−1^), LQC (0.5 ng·mL^−1^), MQC (20 ng·mL^−1^) and HQC (600 ng·mL^−1^) samples. The intra-day precisions of ICA, ICI, and ICII ranged from 7.10% to 10.53%, 5.77%–10.01%, and 4.08%–9.77% at all QC levels, respectively. The inter-day precisions were within 14.64% for ICA, 13.11% for ICI, and 11.55% for ICII. The intra-day accuracy of ICA, ICI, and ICII ranged from −7.71% to 1.76%, −9.39% to −4.86%, and −0.19%–7.28% at all QC levels, respectively. The inter-day accuracy was within −7.23%–1.65% for ICA, −8.81% to −4.27% for ICI, and −0.18%–6.83% for ICII. These results demonstrate that the developed method has satisfactory accuracy and precision for quantifying ICA, ICI and ICII in whole blood samples.

**TABLE 2:** Intra- and inter-day precision and accuracy of the assay (Mean ± SD, n = 5).

Analyte	Concentration (ng·mL^−1^)		Precisions (%)		Accuracy (%)	
	Expected	Measured	Intra-day	Inter-day	Intra-day	Inter-day
ICA	0.25 (LLOQ)	0.24 ± 0.03	10.53	14.64	−3.12	−2.92
	0.5 (LQC)	0.51 ± 0.04	7.17	9.71	1.76	1.65
	20 (MQC)	20.34 ± 1.45	7.12	7.69	1.72	1.61
	600(HQC)	553.73 ± 39.42	7.10	9.05	−7.71	−7.23
ICI	0.25 (LLOQ)	0.24 ± 0.02	10.01	12.19	−4.86	−4.27
	0.5 (LQC)	0.47 ± 0.03	5.99	7.50	−6.40	−5.90
	20 (MQC)	18.1 ± 1.03	5.77	9.84	−9.39	−8.81
	600(HQC)	562.52 ± 37.13	6.71	13.11	−6.25	−5.86
ICII	0.25 (LLOQ)	0.25 ± 0.02	9.77	11.55	−0.19	−0.18
	0.5 (LQC)	0.53 ± 0.03	6.20	5.87	5.57	5.23
	20 (MQC)	21.46 ± 1.09	5.07	6.19	7.28	6.83
	600(HQC)	633.1 ± 25.54	4.08	8.85	5.52	5.17

#### 3.1.4 Extraction recoveries and matrix effects

Protein precipitation extraction was used in this study to extract ICA, ICI and ICII from whole blood. The mean extraction recoveries and matrix effects for this method are shown in Tables 3 and Tables 4, respectively. The mean extraction recoveries ranged from 73.19% to 125.49% for ICA, from 68.29% to 119.67% for ICI, and from 71.23% to 120.80% for ICII. The mean matrix effects ranged from 74.55% to 124.48% for ICA, from 78.03% to 117.14% for ICI, and from 78.30% to 122.90% for ICII. Considering that the extraction recoveries of whole blood samples were reproducible and compatible, the developed method remains sufficient within the detection limit. Moreover, since the matrix effects were within the acceptable range, they were reproducible and did not alter precision or accuracy.

**TABLE 3:** Extraction recoveries of ICA, ICI and ICII in mice whole blood (Mean ± SD, n = 5).

Concentration (ng·mL^−1^)	Extraction recoveries								
	ICA			ICI			ICII		
	Ⅰ(Resp.)	Ⅱ(Resp.)	Ⅰ/Ⅱ (%)	Ⅰ(Resp.)	Ⅱ(Resp.)	Ⅰ/Ⅱ (%)	Ⅰ(Resp.)	Ⅱ(Resp.)	Ⅰ/Ⅱ (%)
0.5 (LQC)	64	51	125.49	35	41	85.37	120	138	86.96
	45	45	100.00	25	31	80.65	134	125	107.20
	44	41	107.32	25	35	71.43	78	91	85.71
	60	49	122.45	28	41	68.29	79	98	80.61
	65	62	104.84	29	42	69.05	148	145	102.07
20 (MQC)	2046	2012	101.69	1,522	1,366	111.42	4,835	4,435	109.02
	2,252	2025	111.21	1,505	1,316	114.36	5,227	4,541	115.11
	2,453	2061	119.02	1,488	1,458	102.06	5,370	4,998	107.44
	1932	1,584	121.97	1,145	1,092	104.85	3,839	3,609	106.37
	1748	1,696	103.07	1,101	920	119.67	3,752	3,106	120.80
600(HQC)	53,650	73,301	73.19	53,926	58,387	92.36	161,425	173,369	93.11
	51,451	65,665	78.35	44,418	46,390	95.75	93,048	126,388	73.62
	66,822	75,962	87.97	38,230	46,905	81.51	127,261	155,735	81.72
	44,009	57,700	76.27	41,599	41,752	99.63	107,454	131,745	81.56
	49,908	54,920	90.87	31,673	40,016	79.15	110,104	154,581	71.23

Ⅰ: Peak area (Resp.) of protein precipitation extraction of LQC, MQC and HQC samples, Ⅱ: Peak area of blank whole blood sample after protein precipitation and addition of compound solution [60%:40% methanol:water (v:v)] containing QC concentration. Extraction recoveries (%) = Ⅰ/Ⅱ.

**TABLE 4:** Matrix effects of ICA, ICI and ICII in mice whole blood (Mean ± SD, n = 5).

Concentration (ng·mL^−1^)	Matrix effect								
	ICA			ICI			ICII		
	Ⅱ (Resp.)	Ⅲ (Resp.)	Ⅱ/Ⅲ (%)	Ⅱ (Resp.)	Ⅲ (Resp.)	Ⅱ/Ⅲ (%)	Ⅱ (Resp.)	Ⅲ (Resp.)	Ⅱ/Ⅲ (%)
0.5 (LQC)	51	67	76.12	41	46	89.13	138	123	112.20
	45	52	86.54	31	30	103.33	125	110	113.64
	41	55	74.55	35	33	106.06	91	95	95.79
	49	56	87.50	41	35	117.14	98	103	95.15
	62	75	82.67	42	41	102.44	145	154	94.16
20 (MQC)	2012	2080	96.73	1,366	1,659	82.34	4,435	5,105	86.88
	2025	2,319	87.32	1,316	1,649	79.81	4,541	5,443	83.43
	2061	2,545	80.98	1,458	1740	83.79	4,998	5,711	87.52
	1,584	1977	80.12	1,092	1,332	81.98	3,609	4,272	84.48
	1,696	1773	95.66	920	1,179	78.03	3,106	3,967	78.30
600(HQC)	73,301	62,925	116.49	58,387	53,353	109.44	173,369	141,070	122.90
	65,665	53,093	123.68	46,390	40,890	113.45	126,388	112,707	112.14
	75,962	61,024	124.48	46,905	48,705	96.30	155,735	142,120	109.58
	57,700	51,254	112.58	41,752	39,996	104.39	131,745	111,011	118.68
	54,920	51,074	107.53	40,016	40,390	99.07	154,581	126,044	122.64

Ⅱ: Peak area of blank whole blood sample after protein precipitation and addition of compound solution [60%:40% methanol: water (v:v)] containing QC concentration, Ⅲ: Peak area of corresponding QC sample prepared with pure water as matrix. Matrix effects (%) = Ⅱ/Ⅲ.

#### 3.1.5 Stability

The processing stability of ICA, ICI and ICII in whole blood was investigated using two parameters: short-term stability and autosampler stability, as shown in Table 5. The analytes were found to be stable in mice whole blood at room temperature (25°C) for up to 6 h, with a relative error (RE) range of −14.26%–8.71%. The autosampler stability was also assessed by placing the extracted quality control (QC) samples in the autosampler and keeping them at 4°C for 24 h, with a reduction of less than 10.91%. All data were within the acceptable range, indicating that ICA, ICI and ICII were stable under routine laboratory conditions. Therefore, no additional procedure was necessary to stabilize the compound for pharmacokinetic studies.

**TABLE 5:** Stability of ICA, ICI and ICII in mice whole blood under various storage conditions (Mean ± S.D., n = 5).

Analyte	Expected (ng·mL^−1^)	Short-time stability 25°C, 6 h		The autosampler stability 4°C, 24 h	
		Measured (ng·mL^−1^)	RE (%)	Measured (ng·mL^−1^)	RE (%)
ICA	0.5 (LQC)	0.53 ± 0.07	6.58	0.44 ± 0.03	−11.70
	20 (MQC)	20.85 ± 2.24	4.25	18.32 ± 0.47	−8.40
	600(HQC)	514.42 ± 29.96	−14.26	521.6 ± 29.32	−13.07
ICI	0.5 (LQC)	0.54 ± 0.09	8.05	0.55 ± 0.03	9.85
	20 (MQC)	20.75 ± 1.99	3.73	20.79 ± 0.89	3.95
	600(HQC)	652.28 ± 7.13	8.71	665.44 ± 13.54	10.91
ICII	0.5 (LQC)	0.52 ± 0.08	3.41	0.49 ± 0.07	−1.82
	20 (MQC)	19.8 ± 2.13	−0.98	21.91 ± 1.01	9.55
	600(HQC)	554.88 ± 43.98	−7.52	596.46 ± 27	−0.59

#### 3.1.6 Dilution reliability

To ensure accurate quantitation, whole blood samples with a high concentration of ICA need to be diluted before processing. Therefore, we measured the dilution reliability of ICA in the whole blood of mice. The results showed that the accuracy and precision of the samples were within 15% after a 50-fold dilution. The established dilution method was found to be accurate and repeatable, and the tested samples met the acceptable criteria outlined in Table 6.

**TABLE 6:** 50-fold dilution reliability for assay of ICA in mice whole blood (Mean ± S.D., n = 5).

Analyte	Concentration (ng·mL^−1^)		RSD (%)	RE (%)
	Expected	Measured		
ICA	600	517.31 ± 7.48	1.45	−13.78

### 3.2 Pharmacokinetic study

The main pharmacokinetic parameters of ICA, ICI, and ICII are presented in Table 7. The mean drug concentration-time profiles of ICA, ICI, and ICII after intravenous administration of ICA at a dose of 15 mg·kg^−1^ are shown, and the mean drug concentration-time profiles of ICII after intragastric administration of ICA at three doses of 30, 90 and 150 mg·kg^−1^ are presented in Figure 3D. After the intravenous administration, a small amount of ICA was converted into metabolites, while after the intragastric administration, most of the ICA was converted to ICII, and ICA and ICI were not detected.

**TABLE 7:** Pharmacokinetic parameters after administration of Icariin in C57 mice (Mean ± SD, n = 5).

Parameters	Unit	Intravenous injection of 15 mg kg^−1^ ICA			Results of ICII after intragastric administration of ICA		
		ICA	ICI	ICII	30 mg kg^−1^	90 mg kg^−1^	150 mg kg^−1^
T_1/2_	h	2.25 ± 2.00	0.47 ± 0.18	2.8 ± 0.84	1.65 ± 0.65	1.53 ± 0.44	2.49 ± 0.55
T_max_	h	0.03 ± 0.00	0.03 ± 0.00	0.03 ± 0.00	2.00 ± 0.00	2.00 ± 0.35	1.80 ± 0.45
C_max_	ng·mL^−1^	13,266.22 ± 5,589.44	187.89 ± 39.17	32.13 ± 10.43	51.68 ± 15.01	146.87 ± 32.41	225.49 ± 20.29
AUC _(0-t)_	h·ng·mL^−1^	1,299.44 ± 480.92	17.08 ± 3.49	8.46 ± 3.08	50.97 ± 19.30	161.16 ± 34.02	273.85 ± 27.82
AUC _(0-∞)_	h·ng·mL^−1^	1,302.03 ± 484.95	17.46 ± 3.64	10.10 ± 2.910	53.80 ± 19.20	162.18 ± 33.95	278.19 ± 28.04
V	L·kg^−1^	32.42 ± 19.68	574.66 ± 121.08	6,254.2 ± 1869.39	1,532.73 ± 869.85	1,346.58 ± 781.71	1980.14 ± 578.30
CL	L·h^−1^·kg^−1^	12.65 ± 3.92	892.88 ± 205.69	1,592.58 ± 480.14	628.09 ± 258.15	578.57 ± 142.79	545.39 ± 59.36

## 4 Discussion

The objective of the current study was to determine the pharmacokinetics of Icariin (ICA) in C57 mice by analyzing micro-whole blood samples collected from the same mouse. Mice are widely used in research to understand human diseases due to their biological similarities, rapid reproductive rates, small size, and low cost. However, due to their small size and low blood volume, sparse or discrete blood sampling is commonly used for pharmacokinetic studies in mice (Raje et al., 2020). To reduce the number of animals used and improve data quality, we successfully collected serial blood samples from the same mouse. We developed a UPLC-MS/MS method to determine ICA and its metabolites, Icariside I (ICI) and Icariside II (ICII), in whole blood using Daidzein as the internal standard (IS). The sample preparation method involved protein precipitation followed by re-dissolution after evaporation to dryness. This method met the 9,012 guidelines (Chinese Pharmacopoeia Commission, 2015). The method had a detection time of only 5.8 min, which is shorter than other methods (Cheng et al., 2015), and the lower limit of quantification of ICA, ICI and ICII were 0.25 ng·mL^−1^, respectively, which was a factor of 2-4 lower than the reported methods (Xu et al., 2007). Using this method, we successfully evaluated the pharmacokinetics of ICA, ICI and ICII after administration of ICA in C57 mice.

After intravenous injection of ICA, the drug concentration-time curve showed a rapid decrease in the concentration of ICA with a T_1/2_ of 2.25 ± 2.00 h. ICA was rapidly transformed into ICI and ICII within 2 min of entering the body. Compared to ICA, the V and CL values of ICI and ICII were substantially higher, and C_max_, T_1/2_ and AUC_0–t_ values were significantly lower, indicating that ICA can be rapidly metabolized into ICI and ICII. According to the AUC_0-t_ comparison, only 1.31% of ICA was transformed into ICI and 0.65% of ICA was transformed into ICII. After intragastric administration, ICA was immediately absorbed and transformed into ICII, and neither ICA nor ICI could be detected. The mean drug concentration-time profiles of ICII after intragastric administration of ICA showed that ICII could be detected from 0.25 h and reached its T_max_ for the three doses (30, 90 and 150 mg·kg^−1^) at 2.00 ± 0.00 h, 2.00 ± 0.35 h and 1.80 ± 0.45 h, respectively. The T_1/2_ for the three doses (30, 90 and 150 mg·kg^−1^) were 1.65 ± 0.65 h, 1.53 ± 0.44 h and 2.49 ± 0.55 h, respectively. The main transformation of ICA in the whole blood after intragastric administration was ICII, whereas ICA remained unchanged when intravenously administered. The poor absorption efficacy of ICA was consistent with the fact that ICA belongs to the disaccharide family and cannot be directly absorbed (Chen et al., 2011). There are two reasons why ICA cannot be detected: firstly, ICA can be decomposed into metabolites by enzymes in the gastrointestinal tract and intestinal microflora after entering the body of mice (Zhou et al., 2013); secondly, the protein binding rate of ICA is 80.05% (YE et al., 1999), and the binding degree is high, which to some extent affects the concentration of free Icariin in the blood. Only free drugs can be bio-transformed in the liver or excreted through the kidney. ICI was undetectable, suggesting that it was not directly absorbed. The vast majority of ICA was transformed into ICII after intragastric administration. As the main active component of ICA metabolite, ICII can suppress Aβ production via promoting the non-amyloidogenic APP cleavage process and markedly decrease the phosphodiesterase-5A (PDE5A) expression to treat Alzheimer’s disease (Yin et al., 2016).

In comparison, Cheng et al. reported the pharmacokinetics of ICA and its metabolite ICII in plasma after intravenous and intragastric administration of 30 mg·kg^−1^ ICA in rats (Cheng et al., 2015). After intravenous administration of 30 mg·kg^−1^ ICA in rats, T_1/2_ and AUC_0-t_ of ICA were 5.33 h and 11,570 h·ng·mL^−1^, while T_1/2_ and AUC_0-t_ of ICII were 1.97 h and 49.95 h·ng·mL^−1^. After intravenous administration of 15 mg kg^−1^ ICA in C57 mice, T_1/2_ and AUC_0-t_ of ICA were 2.25 h and 1,302.03 h·ng·mL^−1^, and T_1/2_ and AUC_0-t_ of ICII were 2.80 h and 10.10 h·ng·mL^−1^. ICA was rapidly converted to ICII in rats and mice, but ICA and ICII were absorbed faster in C57 mice than in rats. After intragastric administration of 30 mg·kg^−1^ ICA in rats, C_max_, T_max_, T_1/2_ and AUC_0-t_ of ICA were 27.20 ng·mL^−1^, 0.25 h, 1.23 h and 10.71 h·ng·mL^−1^, respectively, while C_max_, T_max_, T_1/2_, and AUC_0-t_ of ICII were 29.60 ng·mL^−1^, 2.45 h, 4.36 h and 106.72 h·ng·mL^−1^, respectively. After the intragastric administration of 30 mg·kg^−1^ ICA in C57 mice, the drug concentration of ICA was not detected, while C_max_, T_max_, T_1/2_ and AUC_0-t_ of ICII were 51.68 ng·mL^−1^, 2.00 h, 1.65 h and 50.97 h·ng·mL^−1^, respectively. The conversion rate of ICA to ICII in C57 mice was higher than that in rats, and the absorption rate of ICII in C57 mice was faster. ICA could be detected in rats after intragastric administration of ICA, which is different from the results of this experiment, possibly due to the high binding rate of ICA protein (Cheng et al., 2015).

This study used protein precipitation method to treat micro-whole blood in the same mouse to obtain a complete drug concentration-time curve, and obtained the pharmacokinetics of ICA in C57 mice for the first time. C57 mice as background mice of APP/PS1 transgenic mice participated in this study, and the analytical method and pharmacokinetic results provided a basis for the study of ICA and its metabolisms in APP/PS1 transgenic mice.

## 5 Conclusion

A serial blood sampling technique was developed and used to study the pharmacokinetics of Icariin in C57 mice. A small volume of 30 µL blood was collected from each mouse at multiple time points to generate a complete drug concentration-time curve. A UPLC-MS/MS method was established to determine Icariin and its main metabolites in whole blood of mice using micro-whole blood. The method had a LLOQ of 0.25 ng·mL^−1^ for ICA, ICI, and ICII, respectively, which was a factor of 2-4 lower than the reported methods.

The developed method was used to investigate the pharmacokinetic actions of ICA. The main pharmacokinetic parameters of ICA, ICI, and ICII in C57 mice were studied according to the mode of administration (intragastric or intravenous). After intravenous administration (15 mg·kg^−1^) of ICA in C57 mice, ICI and ICII were detected within 2 min. AUC_0-t_ of ICA, ICI and ICII was 1,299.44 ± 480.92, 17.08 ± 3.49 and 8.46 ± 3.08 h·ng·mL^−1^, respectively. After intragastric administration (30, 90 and 150 mg·kg^−1^) of ICA in C57 mice, ICA was rapidly converted into ICII in mice, and ICA and ICI were not detected. C_max_ of ICII for the three doses (30, 90 and 150 mg·kg^−1^) was 51.68 ± 15.01, 146.87 ± 32.41 and 225.49 ± 20.29 ng·mL^−1^, respectively. T_1/2_ of ICII was 1.65 ± 0.65, 1.53 ± 0.44 and 2.49 ± 0.55 h, respectively, and AUC_0-t_ of ICII was 50.97 ± 19.30, 161.16 ± 34.02 and 273.85 ± 27.82 h·ng·mL^−1^, respectively. The results of this experiment are of great significance for the future preclinical and clinical trials of ICA.

## Data Availability

The raw data supporting the conclusion of this article will be made available by the authors, without undue reservation.
