# Supplementary material for: UPLC-MS/MS method for Icariin and metabolites in whole blood of C57 mice: development, validation, and pharmacokinetics study
Source: Front Pharmacol. 2023 Jul 20;14:1195525. doi: 10.3389/fphar.2023.1195525 (PMC10398387; doi:10.3389/fphar.2023.1195525)
Supplement: Supplementary file 1 [file Table1.DOCX]

**TABLES**

**Table 1.** Linearity for ICA, ICI and ICII in whole blood (n=5)

| Analyte | Batch | ICA | | Linear range |
| --- | --- | --- | --- | --- |
|  |  | Regression equ. | R^2^ |  |
| ICA | 1 | y=0.0237x+0.0109 | 0.9914 | 0.25-800 ng·mL^-1^ |
|  | 2 | y=0.0105x+0.0002 | 0.9925 |  |
|  | 3 | y=0.0169x+0.0002 | 0.9940 |  |
|  | 4 | y=0.0196x+0.0021 | 0.9927 |  |
|  | 5 | y=0.0127x+0.0013 | 0.9925 |  |
| ICI | 1 | y=0.0130x+0.0011 | 0.9933 |  |
|  | 2 | y=0.0095x-0.0011 | 0.9923 |  |
|  | 3 | y=0.0096x+0.0004 | 0.9911 |  |
|  | 4 | y=0.0113x+0.0001 | 0.9928 |  |
|  | 5 | y=0.0081x-0.0003 | 0.9934 |  |
| ICII | 1 | y=0.0442x+0.0067 | 0.9941 |  |
|  | 2 | y=0.0334x+0.0015 | 0.9918 |  |
|  | 3 | y=0.0333x+0.0048 | 0.9918 |  |
|  | 4 | y=0.0384x+0.0066 | 0.9959 |  |
|  | 5 | y=0.0259x+0.0007 | 0.9901 |  |

**Table 2.** Intra- and inter-day precision and accuracy of the assay (Mean ± SD, n=5)

| Analyte | Concentration (ng·mL^-1^) | | Precisions (%) | | Accuracy (%) | |
| --- | --- | --- | --- | --- | --- | --- |
|  | Expected | Measured | Intra-day | Inter-day | Intra-day | Inter-day |
| ICA | 0.25(LLOQ) | 0.24±0.03 | 10.53 | 14.64 | -3.12 | -2.92 |
|  | 0.5(LQC) | 0.51±0.04 | 7.17 | 9.71 | 1.76 | 1.65 |
|  | 20(MQC) | 20.34±1.45 | 7.12 | 7.69 | 1.72 | 1.61 |
|  | 600(HQC) | 553.73±39.42 | 7.10 | 9.05 | -7.71 | -7.23 |
| ICI | 0.25(LLOQ) | 0.24±0.02 | 10.01 | 12.19 | -4.86 | -4.27 |
|  | 0.5(LQC) | 0.47±0.03 | 5.99 | 7.50 | -6.40 | -5.90 |
|  | 20(MQC) | 18.1±1.03 | 5.77 | 9.84 | -9.39 | -8.81 |
|  | 600(HQC) | 562.52±37.13 | 6.71 | 13.11 | -6.25 | -5.86 |
| ICII | 0.25(LLOQ) | 0.25±0.02 | 9.77 | 11.55 | -0.19 | -0.18 |
|  | 0.5(LQC) | 0.53±0.03 | 6.20 | 5.87 | 5.57 | 5.23 |
|  | 20(MQC) | 21.46±1.09 | 5.07 | 6.19 | 7.28 | 6.83 |
|  | 600(HQC) | 633.1±25.54 | 4.08 | 8.85 | 5.52 | 5.17 |

**Table 3**. Extraction recoveries of ICA, ICI and ICII in mice whole blood (Mean ± SD, n=5)

| Concentration (ng·mL^-1^) | Extraction recoveries | | | | | | | | |
| --- | --- | --- | --- | --- | --- | --- | --- | --- | --- |
|  | ICA | | | ICI | | | ICII | | |
|  | Ⅰ(Resp.) | Ⅱ(Resp.) | Ⅰ/Ⅱ (%) | Ⅰ(Resp.) | Ⅱ(Resp.) | Ⅰ/Ⅱ (%) | Ⅰ(Resp.) | Ⅱ(Resp.) | Ⅰ/Ⅱ (%) |
| 0.5(LQC) | 64 | 51 | 125.49 | 35 | 41 | 85.37 | 120 | 138 | 86.96 |
|  | 45 | 45 | 100.00 | 25 | 31 | 80.65 | 134 | 125 | 107.20 |
|  | 44 | 41 | 107.32 | 25 | 35 | 71.43 | 78 | 91 | 85.71 |
|  | 60 | 49 | 122.45 | 28 | 41 | 68.29 | 79 | 98 | 80.61 |
|  | 65 | 62 | 104.84 | 29 | 42 | 69.05 | 148 | 145 | 102.07 |
| 20(MQC) | 2046 | 2012 | 101.69 | 1522 | 1366 | 111.42 | 4835 | 4435 | 109.02 |
|  | 2252 | 2025 | 111.21 | 1505 | 1316 | 114.36 | 5227 | 4541 | 115.11 |
|  | 2453 | 2061 | 119.02 | 1488 | 1458 | 102.06 | 5370 | 4998 | 107.44 |
|  | 1932 | 1584 | 121.97 | 1145 | 1092 | 104.85 | 3839 | 3609 | 106.37 |
|  | 1748 | 1696 | 103.07 | 1101 | 920 | 119.67 | 3752 | 3106 | 120.80 |
| 600(HQC) | 53650 | 73301 | 73.19 | 53926 | 58387 | 92.36 | 161425 | 173369 | 93.11 |
|  | 51451 | 65665 | 78.35 | 44418 | 46390 | 95.75 | 93048 | 126388 | 73.62 |
|  | 66822 | 75962 | 87.97 | 38230 | 46905 | 81.51 | 127261 | 155735 | 81.72 |
|  | 44009 | 57700 | 76.27 | 41599 | 41752 | 99.63 | 107454 | 131745 | 81.56 |
|  | 49908 | 54920 | 90.87 | 31673 | 40016 | 79.15 | 110104 | 154581 | 71.23 |

**Table 4**. Matrix effect of ICA, ICI and ICII in mice whole blood (Mean ± SD, n=5)

| Concentration (ng·mL^-1^) | Matrix effect | | | | | | | | |
| --- | --- | --- | --- | --- | --- | --- | --- | --- | --- |
|  | ICA | | | ICI | | | ICII | | |
|  | Ⅱ(Resp.) | Ⅲ(Resp.) | Ⅱ/Ⅲ (%) | Ⅱ(Resp.) | Ⅲ(Resp.) | Ⅱ/Ⅲ (%) | Ⅱ(Resp.) | Ⅲ(Resp.) | Ⅱ/Ⅲ (%) |
| 0.5(LQC) | 51 | 67 | 76.12 | 41 | 46 | 89.13 | 138 | 123 | 112.20 |
|  | 45 | 52 | 86.54 | 31 | 30 | 103.33 | 125 | 110 | 113.64 |
|  | 41 | 55 | 74.55 | 35 | 33 | 106.06 | 91 | 95 | 95.79 |
|  | 49 | 56 | 87.50 | 41 | 35 | 117.14 | 98 | 103 | 95.15 |
|  | 62 | 75 | 82.67 | 42 | 41 | 102.44 | 145 | 154 | 94.16 |
| 20(MQC) | 2012 | 2080 | 96.73 | 1366 | 1659 | 82.34 | 4435 | 5105 | 86.88 |
|  | 2025 | 2319 | 87.32 | 1316 | 1649 | 79.81 | 4541 | 5443 | 83.43 |
|  | 2061 | 2545 | 80.98 | 1458 | 1740 | 83.79 | 4998 | 5711 | 87.52 |
|  | 1584 | 1977 | 80.12 | 1092 | 1332 | 81.98 | 3609 | 4272 | 84.48 |
|  | 1696 | 1773 | 95.66 | 920 | 1179 | 78.03 | 3106 | 3967 | 78.30 |
| 600(HQC) | 73301 | 62925 | 116.49 | 58387 | 53353 | 109.44 | 173369 | 141070 | 122.90 |
|  | 65665 | 53093 | 123.68 | 46390 | 40890 | 113.45 | 126388 | 112707 | 112.14 |
|  | 75962 | 61024 | 124.48 | 46905 | 48705 | 96.30 | 155735 | 142120 | 109.58 |
|  | 57700 | 51254 | 112.58 | 41752 | 39996 | 104.39 | 131745 | 111011 | 118.68 |
|  | 54920 | 51074 | 107.53 | 40016 | 40390 | 99.07 | 154581 | 126044 | 122.64 |

**Table 5.** Stability of ICA, ICI and ICII in mice whole blood under various storage conditions (Mean ± S.D., n=5)

| Analyte | Expected (ng·mL^-1^) | Short-time stability  25℃, 6h | | The autosampler stability  4℃, 24h | |
| --- | --- | --- | --- | --- | --- |
|  |  | Measured (ng·mL^-1^) | RE (%) | Measured (ng·mL^-1^) | RE (%) |
| ICA | 0.5(LQC) | 0.53±0.07 | 6.58 | 0.44±0.03 | -11.70 |
|  | 20(MQC) | 20.85±2.24 | 4.25 | 18.32±0.47 | -8.40 |
|  | 600(HQC) | 514.42±29.96 | -14.26 | 521.6±29.32 | -13.07 |
| ICI | 0.5(LQC) | 0.54±0.09 | 8.05 | 0.55±0.03 | 9.85 |
|  | 20(MQC) | 20.75±1.99 | 3.73 | 20.79±0.89 | 3.95 |
|  | 600(HQC) | 652.28±7.13 | 8.71 | 665.44±13.54 | 10.91 |
| ICII | 0.5(LQC) | 0.52±0.08 | 3.41 | 0.49±0.07 | -1.82 |
|  | 20(MQC) | 19.8±2.13 | -0.98 | 21.91±1.01 | 9.55 |
|  | 600(HQC) | 554.88±43.98 | -7.52 | 596.46±27 | -0.59 |

**Table 6**. 50-fold dilution reliability for assay of ICA in mice whole blood (Mean ± S.D., n=5)

| Analyte | Concentration (ng·mL^-1^) | | RSD (%) | RE (%) |
| --- | --- | --- | --- | --- |
|  | Expected | Measured |  |  |
| ICA | 600 | 517.31±7.48 | 1.45 | -13.78 |

| Parameters | Unit | Intravenous Injection of 15 mg·kg^-1^ ICA | | | Results of ICII after intragastric Administration of ICA | | |
| --- | --- | --- | --- | --- | --- | --- | --- |
|  |  | ICA | ICI | ICII | 30mg·kg^-1^ | 90mg·kg^-1^ | 150mg·kg^-1^ |
| T_1/2_ | h | 2.25±2 | 0.47±0.18 | 2.8±0.84 | 1.65±0.65 | 1.53±0.44 | 2.49±0.55 |
| T_max_ | h | 0.03±0 | 0.03±0 | 0.03±0 | 2±0 | 2±0.35 | 1.8±0.45 |
| C_max_ | ng·mL^-1^ | 13266.22±5589.44 | 187.89±39.17 | 32.13±10.43 | 51.68±15.01 | 146.87±32.41 | 225.49±20.29 |
| AUC _(0-t)_ | h·ng·mL^-1^ | 1299.44±480.92 | 17.08±3.49 | 8.46±3.08 | 50.97±19.3 | 161.16±34.02 | 273.85±27.82 |
| AUC _(0-∞)_ | h·ng·mL^-1^ | 1302.03±484.95 | 17.46±3.64 | 10.1±2.91 | 53.8±19.2 | 162.18±33.95 | 278.19±28.04 |
| V | L·kg^-1^ | 32.42±19.68 | 574.66±121.08 | 6254.2±1869.39 | 1532.73±869.85 | 1346.58±781.71 | 1980.14±578.3 |
| CL | L·h-1·kg^-1^ | 12.65±3.92 | 892.88±205.69 | 1592.58±480.14 | 628.09±258.15 | 578.57±142.79 | 545.39±59.36 |

**Table 7**. Pharmacokinetic parameters after intravenous and intragastric administration of Icariin in C57 mice (Mean ± SD, n = 5)
